# A Novel Forensic Readiness Framework Applicable to the Drone Forensics Field

**DOI:** 10.1155/2022/8002963

**Published:** 2022-02-28

**Authors:** Fahad Mazaed Alotaibi, Arafat Al-Dhaqm, Yasser D. Al-Otaibi

**Affiliations:** ^1^Department Information System, Faculty of Computing and Information Technology (FCIT), King Abdulaziz University, Jeddah, Saudi Arabia; ^2^School of Computing, Faculty of Engineering, Universiti Teknologi, Skudai, Malaysia; ^3^Department of Computer Science, Aden Community College, Aden, Yemen; ^4^Department of Information Systems, Faculty of Computing and Information Technology in Rabigh, King Abdulaziz University, Jeddah 21589, Saudi Arabia

## Abstract

The Drone Forensics (DRFs) field is a branch of digital forensics, which involves the identification, capture, preservation, reconstruction, analysis, and documentation of drone incidents. Several models have been proposed in the literature for the DRF field, which generally discusses DRF from a reactive forensic perspective; however, the proactive forensic perspective is missing. Therefore, this paper proposes a novel forensic readiness framework called Drone Forensics Readiness Framework (DRFRF) using the design science method. It consists of two stages: (i) proactive forensic stage and (ii) reactive forensic stage. It considers centralized logging of all events of all the applicants within the drone device in preparation for an examination. It will speed up gathering data when an investigation is needed, permitting the forensic investigators to handle the examination and analysis directly. Additionally, digital forensics analysts can increase the possible use of digital evidence while decreasing the charge of performing forensic readiness. Thus, both the time and cost required to perform forensic readiness could be saved. The completeness, logicalness, and usefulness of DRFRF were compared to those of other models already existing in the DRF domain. The results showed the novelty and efficiency of DRFRF and its applicability to the situations before and after drone incidents.

## 1. Introduction

Digital forensics is a significant domain that involves capturing and analyzing cybercrimes. It has many branches: database forensics, IoT forensics, cloud forensics, drone forensics, wireless forensics, malware forensics, mobile forensics, network forensics, and data forensics. These branches have numerous and redundant forensics models, frameworks, approaches, policies, procedures, and tasks. However, the digital forensics domain suffers from the absence of a standardized forensic framework to deal with all these branches. To deal with all cybercrimes, the digital forensics field consists of two stages: proactive forensics and reactive forensics. Proactive forensics refers to forensic readiness before a crime happens; this stage prepares and collects digital evidence to avoid any future risk or disaster. On the other hand, the reactive forensics stage refers to the process after the occurrence of the crime. The main aim of this stage is to identify, capture, preserve, analyze, and document cybercrime. Therefore, all digital forensics branches are categorized under these two stages. Studies on the proactive forensics approach have mainly explored forensic readiness within the context of the ISO/IEC 27043:2015 standard [1–7]. Proactive strategies towards enhancing digital forensics suggest that measures can be implemented within the system under consideration so that relevant and potentially useful pieces of evidence could be collected in a forensically sound manner before the occurrence of a digital incident. Therefore, this is the first study to develop and validate a novel forensic readiness framework applicable to the DRF field using the metamodeling approach. The proposed forensic framework, called Drone Forensics Readiness Framework (DRFRF), consists of two stages: (i) proactive forensics stage and (ii) reactive forensics stage. DRFRF was validated using the qualitative technique (comparison against other models). The results showed that DRFRF is novel and can deal with drone crimes both before and after the crime occurrence.

The rest of the article is structured as follows: Section 2 provides related work; then, Section 3 introduces a methodology.  Section 4 provides a discussion and finally, the conclusion is presented in Section 5.

## 2. Related Work

Several models and frameworks have been proposed for the DRF field in the literature. These models and frameworks have discussed DRF from four perspectives: (1) forensic analysis, (2) nonforensic analysis, (3) forensic framework, and (4) application in the forensic analysis [8]. For example, the authors in [8, 9] discussed how to recover the required evidence in case a drone is investigated under digital forensics circumstances. These studies mainly focused on the wireless forensic aspects; the researchers in [10] were centered on all parts of a drone. However, both highlighted the Linux Operating System and its desirable capacities in collecting evidence on the Linux file system. Remember that drones require an OS to work. In [11], an attempt was made to design a certain tool with the help of Java-FX to be well applied to the visualization of real-time flight control. This tool cannot be implemented directly in forensics; though, it can establish an effective connection between the controller and the drone for data transferring procedures, and it can visualize sensor parameters such as IMU, GPS, and altitude for pilots, hence providing a flight with a high level of safety.

Similarly, in [12], the DJI Phantom 2 Vision Plus was forensically analyzed to answer the following critical question: “Can the flight path of a UAV be reconstructed with the use of positional data collected from the UAV?” In addition, a concise investigation of counterforensic methods was conducted to ascertain if the flight path record could be detected. In another research, a preliminary forensic analysis of the Parrot Bebop was done in [13]. The Parrot Bebop can be named as the only UAV comparable with the Parrot AR Drone 2.0. The researcher in [14] addressed the key challenges in UAV forensic analyses and then carried out his investigation on two separate parts: UAV and flight controller. The flight-related data were retrieved from the device in the form of “.pud” files. Moreover, a new “.pud” file was formed at each session between the controller and UAV. At the opening point of each “.pud” file, a set of metadata was explored, which consisted of the serial number of the UAV, the flight date and time, the model of the flight controller, and the flight controlling application. Then, an attempt was made to identify the videos/images recorded by the UAV's onboard camera. The images preserved the EXIF data that contained the latitude/longitude coordinates of the places from which the images were taken. The ownership can be established only when the UAV and controller have been seized through the identification of the device serial number.

The authors in [10] attempted to generally review DRF with the use of DJI Phantom 2. They carried out breakdown analyses of the hardware and software components of the drone and discussed the ways they can be applied to the implementation of DRFs. Their findings succeeded in the establishment of a belief in the persistence and scope of DRFs. Besides, this research provides a good opportunity for scrutinizing deeper into this concept and improving it. Moreover, the researchers in [14] worked on integrating the visualizing data retrieved from drones and a nonforensic approach. This study was carried out on the Parrot AR Drone 2.0. With their self-designed application, the log parameters from flight data were visualized, although the evaluation was performed on only a small number of drones. In [15], the authors analyzed the susceptibilities and uses of drones and their relationships with cybersecurity-related issues. The findings confirmed that in cases where drones are hacked and misused by opponents, it could lead to considerable threats or ramifications. Their research mainly tested the benefits of applying drones to many situations, from using them as children's toys to applying them as weapons for mass destruction.

A forensic framework comprising 12 phases was designed in [16] to introduce a new approach through which UAVs can be investigated systematically. They conducted extensive tests on five commercial UAVs, including the Parrot AR Drone 2.0, to identify and understand the relationships among different components. Moreover, an experiment was carried out for the validation of the proposed framework. Each UAV involved in the testing was modified by the removal and/or addition of some of its components. It was done mainly to check whether the framework encompassed all of the different elements in any basic commercial UAV and also to test its applicability to a comprehensive UAV analysis. The authors concluded that the absence of law enforcement training processes in UAV is a key issue that hinders the effective mitigation of attacks. Any of the five UAVs were not subjected to forensic analyses; though, a valuable framework was finally provided, which can help scholars to examine and analyze stages. The first wide-ranging analysis of the DJI Phantom 3 Standard was carried out in [17]. In that study, a forensically sound open-source Drone Open-Source Parser (DROP) tool was also developed. The underinvestigation UAV was flown to two different sites. Afterward, the data acquired were divided into three parts: controller, drone, and phone/tablet. Ultimately, two files of interest were explored: (1) the “.dat” files generated by the UAV and (2) the “.txt” files generated by the DJI GO application. These files were decrypted and decoded; then, the flight information related to Wi-Fi connections, GPS locations, flight status, remote control, motors, etc., were extracted. After the analysis of the acquired data and understanding the proprietary file structures, the DROP tool was developed to analyze the evidentiary files.

Findings reported in [17] showed that if a UAV is turned on, the integrity of the data kept on its internal storage could be impacted. A new “.dat” file is generated each time the UAV is turned on. Moreover, it was found out that in case the SD card is at or near its full capacity, turning on the UAV causes the immediate removal of the oldest data in a way not to be coverable later. As stated in [17], although their research offered an appropriate point to start UAV forensic analysis, further research is required to cover the broad range of UAVs obtainable presently. The authors in [18] provided a comprehensive discussion regarding the ways the GPS coordinates can be applied as location evidence when investigating the crimes committed using drones. They attempted to extract the system logs. They also made a visualization of GPS coordinates on maps, where web-based third-party platforms were employed to plot the flight path.

In another project [16], a forensic model was introduced to determine and authenticate different drone components that can be employed in committing unlawful deeds. The study was centered on the analysis of physical evidence gathered by investigators from the crime scene along with GPS-related data and any multimedia found on board. The research was carried out on five commercial drones together with their components once seized at crime scenes. A key challenge in lowering drone attacks is the shortage of law enforcement training processes in this field. In another study, the researchers [19] made their attempts to find out the correlation of the flight data amongst the drone, SD card, and mobile phone. The establishment of a link between the drone and the suspect could facilitate criminal investigations. Applying specific software to personal UAV devices can provide a plethora of digital artifacts from GPS timestamps and waypoints, the number of satellites connected, barometer, roll, pitch, distance, azimuth, battery status, video, and photos. In [20], the researchers analyzed the essential major log parameters of the autonomous drone and suggested the use of comprehensive software architecture related to DRFs with preliminary results. The authors expected their proposed software to make available a user-friendly graphical user interface (GUI) on which users would be capable of extracting and examining the onboard flight information. They expected to have a contribution to the forensic science community by proposing a tool applicable to investigations on drone-related crime cases. As stated in [21], open-source tools such as CsvView and ExifTool have been employed by several scholars to extract artifacts from mobile applications of drones with the use of mobile forensic techniques. In that study, Kali (which is a Linux distribution) and Windows were employed as forensic workstations to carry out the required forensic analyses on two drones, DJI Phantom 3 and A.R Drone. The open-source tools, e.g., Geo-Player, have been applied mainly to the visualization of flight path data. Because of the nonexistence of an appropriate build environment that includes configuration tools, a package manager, and a compiler in the UAV system, this option needs to extensively change the data that exist in the UAV. Therefore, it was stopped in favor of the logical level acquisition. It was done by mounting a forensic mass storage device onto a UAV; then, files were completely copied from the mounted “/data” partition with the use of the “cp” command. Digital forensics was also applied in [9] to the Parrot A.R Drone 2.0. In that study, several general facts and file formats were discussed, and the flight path was thoroughly visualized with the help of Google Earth. That approach was found with a high focus upon general technical descriptions of a drone with a forensic perspective. In another research [8], in-depth forensic analyses were applied to the Parrot AR Drone 2.0, its GPS Edition, and its outlying components, i.e., the flight recorder and flight controller.

In [22], the researchers attempted to explore the difficulties that may appear in the course of forensically analyzing UAV/drones. To this end, they decided to examine and evaluate the currently used forensic guidelines regarding their efficiency when applied to DRFs analyses. After that, the authors offered their own set of guidelines in this regard and, to end with, they explained the way their guidelines can be effectively implemented when analyzing a drone forensically. As a case study, DJI Phantom 3 drone was used. One of the most important limitations in UAV forensics is the absence of already-confirmed forensically sound tools, which indeed offers a direction for future research. For instance, the next logical step would be creating various parsing tools with the capability to analyze original data and provide legible and dependable information. Moreover, UAVs will have the required capacity to be well integrated with radio communication services in the future.

The authors in [23] proposed an architecture using the Id-Based Signcryption to guarantee the authentication process and privacy preservation. First, the important elements on which the architecture relies were defined. Afterward, the interaction between these elements was examined to understand how the process works. Then, the proposed authentication scheme was explained in detail. As a result, they used the RFID tags to track drones and the temporary identity for the purpose of privacy preservation. A simulation was conducted to calculate the average renewal of temporary identity by varying the time and the drones' speed.

In [24], a captured UAV was analyzed under forensic conditions. Security forces may capture a suspected UAV with the use of a shotgun (or any other applicable technique), or it may be a device that has crashed into private properties. When a UAV is to be subjected to forensic investigations, there is a need to identify its software/hardware modules. Then, it is necessary to collect available evidence, provide the chain of custody, and analyze the media/artefact loaded on the device. On the other hand, the illegitimate use of UAVs, which is increasingly occurring, shows a legal loophole that exists in the currently applied aviation regulations. This has, consequently, led to the shortage of information and prevailing standards on how the UAV incidents could be investigated. Conversely, a study in [25] explored the potential cyber-physical security threats and attempted to address the existing challenges that could be attributed to UAV security before UAVs become the prevailing vehicles in future smart cities. In addition, the authors suggested a method applicable to the investigation of large-scale cyber-security attack vectors of such systems based on four categories of systems, which are of high importance to UAV operations. Moreover, they explained their impacts in detail and the effective ways to counter such attacks. In another project, arbitrary software was designed and applied in [26] to a locked target to gain access to interior sensors and logs of the device using the neutralization and hardening strategies to predict the effectiveness.

In [24], an inclusive-based framework was proposed for drone forensic analysis, involving both physical and digital forensics. In the case of physical forensics, a model was created with the capability to investigate drone components right at the crime scene. The framework had enough proficiency to be applied to the postflight investigations of the activities of the drone. Moreover, the authors designed a powerful application that could be implemented in digital drone forensic analysis, centering mainly upon the analysis of the drones' critical log parameters through a GUI developed with the help of JavaFX 8.0.

In another research [27], a new Distributed, Agent-based Secure Mechanism was proposed for IoD and Smart grid sensors monitoring (DASMIS) scheme. It was designed to run over a hybrid of peer-to-peer (P2P) and client-server (C/S) network architecture with reduced protocol overheads for immediate and bandwidth-efficient communication. In this system, each node is loaded with an initial status and equipped with a python-based agent that is capable of scanning and detecting burned in read-only node-IDs, Node IP Address, node MAC address, system calls made, installed applications, all running system programs and applications, and modifications. Additionally, it performs data encryption and hashing and reports changes to other peer nodes and the server sitting in the C&C center. The agent securely authenticates nodes, enciphers the communication, and authorizes internode access. It prevents and detects attacks such as masquerading, modification, and DoS attacks.

Furthermore, the authors in [28] conducted a study aimed at giving help to whoever is tasked with the generation and analysis, validation, and/or optimization of data to trace evidence recovery. For this purpose, the authors elaborated the approach used to solve this problem based on the target fiber retrieval context using self-adhesive tapes.

Moreover, in [29], the researchers attempted to adapt digital forensic processes capable of improving the drone incident response plan by implementing the digital forensic analysis process. More detailed information was provided regarding the developed Drone Forensic and Incident Response Plan (DFIR) in that study. The findings showed that the Federal Aviation Administration (FAA) can update the requirements of its Unmanned Aerial Systems (UAS) based on two classifications of UAS. They also comprehensively reviewed the related literature and concluded that it lacked studies focusing upon incident responses and forensic analysis frameworks developed specifically for remotely piloted aerials systems. For that reason, the authors made an effort to fill the gap.

The electromagnetic watermarking concept was introduced in [30] as a technique that exploits the IEMI impacts for embedding a watermark into civilian UAVs with the aim of performing forensic tracking.

In [31], the authors surveyed a small sample of aircraft accident investigators and digital forensics investigators and examined their use of forensics frameworks to conduct forensic investigations on drones. The data analyses that were carried out with the use of the chi-square test of independence did not reveal any considerable connection between the groups of respondents' drone investigations and the methods used to conduct UAS forensics.

In [32], drone attacks were discussed from a different perspective. Their study was mainly aimed at identifying where the SDR board is (or could be) applied to the implementation of an attack and/or a countermeasure so that current and future risks could be highlighted. As a result, their analysis was mainly centered on two facets one of which was related to targets of the attacks and the other one to the direction of the attacks. There may be more than one target, which offers multiple possible countermeasures. Targets may include the sensor (mainly GPS), telemetry, remote telecontrol, the embedded software, the physical signature (optical, audio, infrared, electromagnetic, and radar), and/or cognitive channel (cognitive scrambling and stealthy communication). The attacks may be directed from ground to drone, or vice versa, or even from a drone to drone.

The researchers in [33] proposed an innovative method for quickly and accurately detecting whether a drone is flying or lying on the ground. Such results are obtained without resorting to any active technique; rather, they are achieved through just eavesdropping on the radio traffic and processing it through standard machine learning techniques. According to the findings reported in [33], with effective classifying the network traffic, a drone's status can be properly detected with the help of the widespread operating system of ArduCopter (e.g., several DJI and Hobbyking vehicles). Additionally, a lower bound was formed upon the detection delay at the time of applying the above-noted methodology. The proposed solution was capable of discriminating against the drones' state (steady or moving) with roughly 0.93 SR in almost 3.71 seconds.

In [34], the security susceptibility of two drones, namely Eachine E010 and Parrot Mambo FPV, was evaluated. The former drone was found vulnerable to Radio Frequency (RF) replay and custom-made controller attacks, whereas the latter was found susceptible to deauthentication and FTP service attacks. The authors provided a full discussion on both the security susceptibilities of the above-mentioned UAVs and the potential countermeasures that can be taken into action to improve the resilience of UAVs against probable attacks.

The overall legal process to gather and examine any drones from the crime scene and examine inside the lab has been discussed by [35].

Also, [36] proposed a model to collect and document digital data from the flight artifacts and the related mobile devices to help the forensic examination of two common drone systems: the DJI Spark and Mavic Air.

The review of the literature revealed that the DRF field lacks a forensic readiness framework to structure, organize, and unify the DRF field from a readiness perspective. Thus, this study proposes a comprehensive readiness forensic framework applicable to the DRF field.

## 3. Methodology

**Figure 1:**
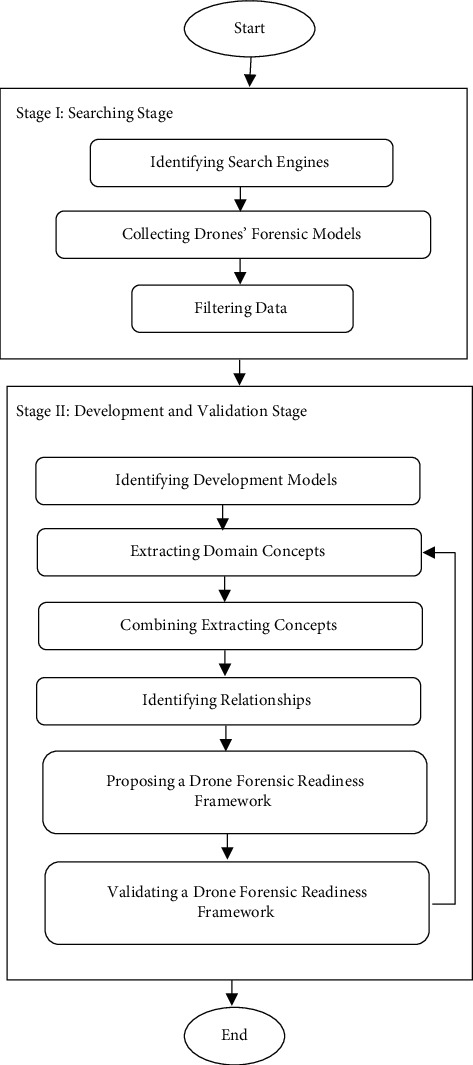
Metamodeling approach [38].

This paper adopted a Design Science Research (DSR) method to develop a drone forensic readiness framework. DSR is a method used to generate original and insistent objects for a particular problem area that allows analytics to be studied [37]. For this research, the metamodeling approach was adopted from [38]. It consists of two stages as shown in Figure 1: searching stage and development and validation stage.

Stage I: searching stage: this stage involves conducting a literature review and collecting data. It consists of three steps:

1. Identifying Search Engines: seven common search engines are used in this study to collect data: IEEE Explorer, Web of Science, Scopus, Springer, ACM, Science Direct, and Google Scholar.
2. Collecting drone forensic models: to collect data, the authors identified keywords (“Drones Forensics,” “Drone Forensics + Model”). Based on the keywords, 132 articles were collected from the literature, as shown in Table 1.
3. Filtering data: regarding the time scope, the search was confined to the studies published between 2000 and January 2021. For the purpose of the present paper, documents such as research articles, conference papers, dissertations, books, and book chapters were considered, and the other types of documents were excluded. In addition, the duplicates and screening of the topic and abstracts were ignored. Table 1 summarizes the details of the search protocols employed in this study. Finally, 29 out of 132 articles were identified to be completely focused upon regarding the topic of DRFs processes and technology perspectives in this field.

**Figure 2:**
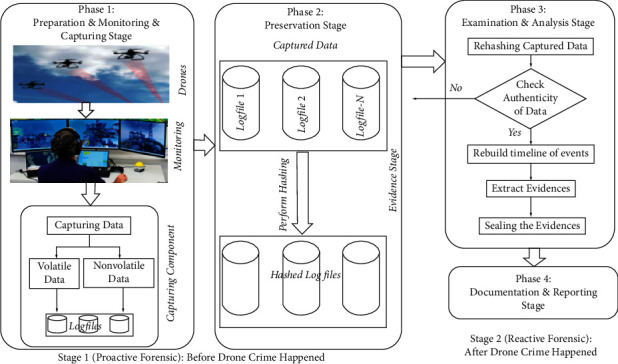
Drone forensic readiness framework (DRFRF).

Stage II: Development and Validation Stage: this stage involves developing and validating DRFRF. It consists of several steps:

1. Identifying the development models: this step aims to identify the development and validation models used to develop and validate the forensic readiness framework.  Table 2 displays the development and validation models.
2. Extracting common concepts and processesCommon processes and concepts were execrated from 32 identified models. The extraction criteria were adopted from [47]: the processes and concepts should be extracted from the model's text body or the flowchart, and the concept or processes must have a definition or activities. Irrelevant processes or concepts were excluded. Thus, 150 common concepts and processes were extracted, as shown in Table 3.
3. Combining extracted processes and conceptsthe common concepts or processes with similar meaning or functioning regardless of their names or synonyms were combined into the same category, as presented in Table 3. Based on the techniques described above, 150 common concepts were categorized into 32 groups. Each group has similar concepts and processes, either in semantic meaning or functional meaning. The common concepts or processes were selected for each group based on frequency [48]. The common concept or process with a higher frequency in the categorization was selected as a common concept, as shown in Table 3.
4. Identifying relationshipsthis step identifies the relationships among proposed drone forensic processes and concepts. A survey of drone forensic models showed various UML relationships amongst the concepts and processes that were common among all such models. Three kinds of common UML relationships were discovered: Association, Specialization/Generalization, and Aggregation.
5. Proposing a drone forensic readiness framework:  the relationships identified in the above step were used to create the drone forensic readiness framework applicable to the drone forensics field (see Figure 2). It consists of two stages: before the occurrence of drone crime (proactive forensics stage) and after the occurrence of drone crime (reactive forensics stage).
6. *Stage 1: Before the Occurrence of Drone Crime.* This stage represents the main stage of the proposed framework. The main aim of this stage is to monitor and capture the whole drones' activities before any crime or incident happens. It is called the proactive forensics stage. It consists of two phases: the monitoring & capturing phase and the preservation phase.
7. *Monitoring and Capturing Phase*. The purpose of this phase is to observe and secure the flight paths of the drone and capture the whole streaming activities (e.g., photos, GPS data, and records). For security purposes, the monitoring component uses a firewall to filter both incoming and outcoming wireless traffic. “Filtering” is defined as the process of controlling access by examining all the packets based on the content of their headers. However, a firewall cannot detect all the misconduct data since some laptop/mobile devices may make their identities unclear to appear as legitimate users of the network. For that reason, our proposed framework employs a component called the Capture Component (CC), which records or logs all the monitored data sent from the drone. CC gathers all the volatile and nonvolatile data monitored to gather potential digital evidence. Each drone has its associated CC that captures/logs the data passing through that laptop/mobile. CC captures/logs the data in log files, as depicted in Figure 2. These log files are working in a circular manner and archive mode to avoid overwritten log files. For example, if the current log file is full, CC will move to the next log file; however, if the current log file is the last one, CC will use the archive mode technique to archive all the log files to avoid loss of evidence. Finally, CC sends the accumulated data logs to the preservation phase to create a hash value for each log file sent to the hashing storage area to preserve evidence.
8. *Preservation Phase*. The primary goal of evidence preservation is to ensure that absolutely no changes are made to the captured/logged data after collection [49]. Figure 1 demonstrates how the log files are preserved in the proposed framework. The Evidence Store (ES) stores all the captured data received from various CCs. In general, ES acts as a central storage area for all the data captured by CC. ES logs the data in chronological order. These data are stored according to CC from which the drone was monitored. It is worth noting that the data stored in ES are needed for analysis purposes only. Analysis of these data will only occur if a particular incident has been reported on the drone, which needs to be investigated. The hash values of the log files are created in the “perform hashing” to hash the whole captured logfiles. Our proposed framework adopts the MD5 and SHA-1 hashing techniques. Hashing is a mathematical function that creates a unique fixed-length string from a message of any length. The result of a hash function is a hash value, sometimes called a message digest. It is worth noting that the hashed blocks of data will only be used to check that the logged data on ES has not been altered during the course of a digital forensic investigation. Preserving the integrity of digital evidence is an absolute requirement of the digital forensic process.
9. *Stage2: After the Occurrence of Drone Crime*. This is the second stage of the proposed framework. It involves conducting a normal digital forensic investigation process to reveal the evidence of the drone crime. It is called the reactive forensic stage. It consists of two processes: examination and analysis process, and documentation and reporting process. The examination process is used to check the authenticity of the gathered data against any tampering through rehashing the captured data. Thus, if the captured data are not authentic, the investigation team should return to the preservation stage to take another original copy. If the captured information is correct and has not been tampered with, the data will move to the analysis phase. The main purpose of the analysis phase of the proposed framework is to mine and extract the data from ES to come up with evidence that can associate a particular adversary with a criminal activity committed on the drone device. The analyzed data are next passed on to the documentation and reporting phase. Although it is not within the scope of this study to discuss data mining in detail, the use of data-mining techniques should not be overlooked during the process of conducting a digital forensic investigation.
10. During the reporting phase, the final evidence is prepared for the entire digital forensic investigation. The data are used by cyber forensic experts when they testify in a court of law that an intruder should be found guilty based on the evidence that they have gathered in their digital forensics investigation. The prosecutor in a court of law has to decide whether the intruder is guilty or not, based on the evidence presented by the cyber forensic experts concerned.
11. *Validating Drone Forensic Readiness Framework*. This is the sixth step of the development and validation process of the drone forensic readiness framework. It is used to validate the proposed framework's completeness, logicalness, and usefulness through a validation technique, namely a comparison of its performance with other models [50]. This comparison aims to identify any missing concepts in the proposed framework and ensure it has sufficiently broad coverage.  Table 4 shows the results of the comparison between the proposed framework (DRFRF) and the existing DRF models. It is very clear that DRFRF is a comprehensive framework and can work in both forensic perspectives, i.e., proactive forensics and reactive forensics.

## 4. Discussion

Through this study, DRF field has suffered from lacking a forensic readiness framework to structure, organize, and unify the DRF field from a readiness perspective, as revealed previously in Section 2. Therefore, this study proposed a comprehensive forensic readiness framework for DRF field. To develop the DRFRF, the Design Science Research (DSR) has been adapted from [37]. Furthermore, the sequence of the processes to follow, particularly for first responders, in a drone-related crime/incident is clearly defined in Stage I of the proposed framework DRFRF. These phases of the framework can be extended to align with the digital forensic readiness phase of the ISO/IEC 27043 standards [26]. Forensic readiness could introduce a standardized approach to potential evidence reliability and extraction before incident occurrence (premortem). Therefore, the phases of the preincident response of the proposed framework DRFRF can be further extended to accommodate organization preparedness against drone downtime while providing reliable content that could otherwise have been lost. An example of this assertion is the monitoring and capturing of volatile data of drone devices. The integration of a methodical approach towards potential evidence identification, collection, and storage in a preincident can be used to address the problem of volatile and nonvolatile evidence preservation. Another core fundamental composition of the proposed DRFRF is the integration of forensic soundness into drone incident investigation. The forensic soundness assurance can provide a reliable corroborative substance, beyond any reasonable doubt, given that the chain-of-custody and chain-of-analysis can be proven at any requested time. Furthermore, the integrity and reliability of any potential evidence are ensured within the preincident and during incident response processes. The integrated framework of the proposed DRFRF is further presented in Figure 2. The output from Stage I is primarily defined as the input to Stage II where chain-of-custody and chain-of-evidence are ensured, respectively. Often, the postincident process is relegated to an afterthought which, potentially leads to a repeated drone incident. Therefore, the proposed framework can be defined as a comprehensive framework that could be used to preempt, prepare for, and prevent a drone incident occurrence.

Without discounting the aforementioned capabilities of DRFRF, the authors of this paper take a step to explore the advantages of DRFRF that supersedes the existing models and the limitations. It is important to note that the limitations that have been identified have carefully been analyzed and positioned to be relevant for inclusion as future work beforehand.

The DRFRF has been juxtaposed as a comprehensive framework that has major inclusion and integration of processes and concepts that have been suggested by existing drone investigation models. While it is important to acknowledge that these models have offered very significant insights towards the development of DRFRF, we put across one core advantage that DRFRF holds. DRFRF is able to cover preincident preparation that has explicitly been presented at a readiness phase. This phase not only is able to shorten the process of conducting an investigation in drones but also saves time due to the availability of forensic evidence when needed. Additionally, the scope of the major phases in the proposed DRFRF (monitoring & capturing phase, and preservation phase, examination and analysis phase, documenting and reporting phase) have been described well based on their functionalities, where DRFRF hold an advantage of leveraging the prescribed guidelines for information technologies, incident investigation techniques, and processes that explicitly are adapted verbatim from ISO7IEC 27043. Next, the DRFRF has room for further integration, which means it is easy to incorporate other suitable processes because of how the different phases have been classified and as a result, the DRFRF activities accept other processes that can be deemed as essential during integration.

At the time of writing this paper, there currently does not exist specific guidelines or standards that address incident response categorically and as such, incident response can only be encapsulated in ISO/IEC 27043 investigative process classes from a generic perspective. This is a current limitation of this framework. However, an inclusion or adoption of these (standardized) guidelines will be inevitable.

## 5. Conclusion

Drone forensics has grown tremendous care from academics working in this field. Drone Forensics (DRF) is a significant field that encompasses the investigations for identifying and discovering drone crimes. Several models and techniques have been proposed for the DRF field. These techniques and models use the interior logs of devices and their controllers to recognize any malicious action. They can duplicate the flight routes that can be used by experts during forensic investigates. The verification and security of drones have also been improved to avoid intrusion. However, the literature lacks a standardized forensics model/framework to deal with different drone crimes. Therefore, this study provided a novel forensic readiness framework that can be applied to the DRF field to deal with drone crimes from both preincident and after-incident perspectives. The proposed DRFRF consists of two stages: (I) proactive forensics stage and (II) reactive forensics stage. The production from Stage I is mainly well-defined as the input to Stage II where chain-of-custody and chain-of-evidence are guaranteed correspondingly. Frequently, the postincident process is referred to as an afterthought which, theoretically, leads a frequent drone crime. Thus, the proposed framework can be defined as a comprehensive framework that could be used to preempt, prepare for, and prevent a drone incident occurrence. The proposed framework DRFRF was validated through a comparison with other existing models. To demonstrate the effectiveness of the proposed DRFRF, a real scenario is required; thus, the future work of this study will focus on the implementation of the DRFRF in a real case.

## Figures and Tables

**Table 1:** Results of search engines.

Database search engines	Number of drone forensic-related articles
Web of Science	10
Scopus	20
IEEE Explore	5
Springer Links	6
Google Scholar	80
ACM	1
Science Direct	10
**Total**	**132**

The bold values mean the total articles which were collected from search engines.

**Table 2:** Development and validation models.

Id	Models references	Year	Authors	Focuses
1	[11]	2015	Mhatre et al.	A forensic examination of the flight path reconstruction method for DJI Phantom 2 Vision Plus.
2	[13]	2016	Horsman	Investigation and analysis of both the DJI Phantom II and DJI Phantom III model UAVs.
3	[15]	2016	Mohan	Testbed model of evidence acquisition from UAVs
4	[10]	2016	Kovar et al.	Preliminary digital forensic analysis of Parrot Bebop UAV (capable of 1080p HD footage and 14 megapixels still images, a 2.4 GHz or 5 GHz Wi-Fi band, s, flight distances can extend beyond 2000 m and to a maximum altitude of 150 m).
5	[39]	2016	Maarse et al.	Development of visualization tool for drone analysis.
6	[14]	2016	Procházka	Drones vulnerabilities
7	[18]	2017	Prastya et al.	Drone forensic framework: Sensor and data identification and verification. Specifically, this research analyzes the architecture of drones and then proposes a generic model that is aimed at improving digital investigation.
8	[16]	2017	Jain et al.	DROP (DRone Open-source Parser) your drone: Forensic analysis of the DJI Phantom III.
9	[17]	2017	Clark et al.	Mainly Forensic Analysis of Unmanned Aerial Vehicle to Obtain GPS Log Data as Digital Evidence. This has been achieved through Digital forensic evidence extraction through the simulation of a UAV scenario that explicitly uses drones.
10	[40]	2017	Bucknell and Bassindal	An investigation into the effect of surveillance drones on textile evidence at crime scenes.
11	[19]	2017	Llewellyn	Drone Forensic Investigation: DJI Spark Drone as A Case Study.
12	[21]	2017	Barton and Azhar	Autonomous Arial Vehicles in Smart Cities: Potential Cyber-Physical Threats.
13	[41]	2017	Renduchintala et al.	An agent-administrator-based security mechanism for distributed sensors and drones for smart grid monitoring.
14	[9]	2018	Bouafif et al.	Drone Forensic Analysis Using Open-Source Tools in The Journal of Digital Forensics, Security and Law.
15	[42]	2018	Roder et al.	Drone Forensics: Challenges and New Insights.
16	[22]	2018	Maune	Unmanned aerial vehicle forensic investigation process: DJI Phantom 3 drone as a case study.
17	[23]	2018	Benzarti et al.	Unlocking the Access to the Effects Induced by IEMI on a Civilian UAV.
18	[43]	2018	Gülataş and Baktır	Unmanned Aerial Vehicle Digital Forensic Investigation Framework.
19	[25]	2018	Dawam et al.	Privacy preservation and drone authentication using ID-Based Signcryption.
20	[26]	2018	Esteves et al.	A comprehensive micro unmanned aerial vehicle (UAV/Drone) forensic framework.
21	[44]	2018	Shi et al.	Antidrone system.
22	[45]	2018	Guvenc et al.	Techniques of detecting and tracking UAV.
23	[46]	2018	Ding et al.	Amateur Drone Surveillance Systems.
24	[24]	2019	Renduchintala et al.	Drone Forensics: Digital Flight Log Examination Framework for Microdrones.
25	[27]	2019	Fitwi et al.	The effect of tape type, taping method, and tape storage temperature on the retrieval rate of fibres from various surfaces: An example of data generation and analysis to facilitate trace evidence recovery validation and optimization.
26	[28]	2019	Jones et al.	Drone Disrupted Denial of Service Attack (3DOS): Towards an Incident Response and Forensic Analysis of Remotely Piloted Aerial Systems (RPASs).
27	[29]	2019	Salamh and Rogers	Electromagnetic Watermarking: exploiting IEMI effects for forensic tracking of UAVs.
28	[30]	2019	Esteves	An Approach to Unmanned Aircraft Systems Forensics Framework.
29	[31]	2019	Esteves et al.	Detecting Drones Status via Encrypted Traffic Analysis.
30	[32]	2019	Le Roy et al.	Assessing and Exploiting Security Vulnerabilities of Unmanned Aerial Vehicles.
31	[33]	2019	Sciancalepore et al.	Risk assessment of SDR-based attacks with UAVs.
32	[34]	2020	Lakew Yihunie et al.	Forensic analysis of the Parrot AR Drone 2.0 GPS Edition and its peripheral components.

**Table 3:** Common concepts and processes.

No.	Propose common processes and concepts	Candidate concepts and processes	Frequency
1	Monitoring and capturing	Monitoring and capturing	3
		Seizure	1
2	Data Acquisition	Gathering evidence	1
		Data acquisition	3
3	Intruder Activity	Intruder's transactions	1
		Intruder activity	2
		Malicious transaction	1
4	Data Collected	Data collected	8
		Acquired data	1
5	Reconstruction	Reconstructing log events	1
		Reconstruction	5
		Reconstruction event	1
		Reconstructing	1
6	Hashing	Hashing	4
7	Examination	Examination	5
8	Backup	Backup	5
9	Preservation	Preservation	4
10	Investigation Team	Investigation Team	9
		Forensic examiner	1
		Examiner	1
11	Integrity	Evidence integrity	1
		Integrity	2
12	Source	Resources	1
		Source	5
13	Evidence	Evidence	6
14	Drone Incident	Event	4
		Drone Incident	5
15	Hashed Value	Hashed Value	3
16	Rehashing	Rehashing	3
17	Log File	Log file	8
		database log file	1
18	Incident Responding	Incident response	1
		Incident responding	1
19	Drone	Drone	7
		UAV	5
20	Court	Court	5
		Court of law	2
21	Live Response	Live response	3
22	Forensic Technique	Forensic Techniques	2
		Investigation extraction methods	1
23	Timeline	Timeline	5
24	Interview	Interview	2
25	Volatile Artefact	Volatile artefact	2
26	Nonvolatile Artefact	Nonvolatile Artefact	2
27	Decision	Decision	2
28	Report	Forensic report	1
		Report	2
		Final forensic report	1
29	Artefact	Artefacts	3
30	Live Acquisition	live acquisition	2
31	Dead Acquisition	Dead acquisition	2
32	Hybrid Acquisition	Hybrid acquisition	2
**Total**			**150**

Bold shows total of common process and concepts.

**Table 4:** Comparison between the exiting DRF models and DRFRF.

Proposed DRFRF	Existing DRF models																														
	[13]	[15]	[11]	[10]	[39]	[14]	[18]	[16]	[17]	[40]	[19]	[24]	[9]	[42]	[22]	[23]	[43]	[21]	[25]	[26]	[41]	[27]	[28]	[29]	[30]	[32]	[33]	[34]	[44]	[45]	[46]
Stage1 proactive forensics	✘	✘	✘	✘	✘	✘	✘	✘	✘	✘	✘	✘	✘	✘	✘	✘	✘	✘	✘	✘	✘	✘	✘	✘	✘	✘	✘	✘	✘	✘	✘
Stage 2: reactive forensics	✓	✓	✓	✓	✓	✓	✓	✓	✓	✓	✓	✓	✓	✓	✓	✓	✓	✓	✓	✓	✓	✓	✓	**✓**	**✓**	**✓**	**✓**	**✓**	**✓**	**✓**	**✓**

## Data Availability

All the data used to support the study are included within the article.
